# Supplementary material for: Association of triglyceride-glucose index with the risk of prostate cancer: a retrospective study
Source: PeerJ. 2023 Nov 7;11:e16313. doi: 10.7717/peerj.16313 (PMC10637243; doi:10.7717/peerj.16313)

# flowsheet

The first affiliated hospital of Xinjiang medical university is the largest medical institutions in Xinjiang region, urology specially set up prostate cancer follow-up center has for prostate cancer patients established a perfect medical records and follow-up system, the first affiliated hospital of Xinjiang medical university urology surgery is Xinjiang urinary male germ line disease clinical research center and China national center for prostate cancer quality control. In this study, there were 1,303 prostate cancer patients. In the control group, we selected 22,611 patients from the same hospital. We all set strict inclusion and exclusion criteria. Since the TyG index can be affected by blood glucose, blood pressure, and some underlying diseases, we excluded all people with hypertension and hyperglycemia from the exclusion criteria.The case group was screened for primary prostate cancer patients (N=203). Hyperglycemia and loss to follow-up (n=67) were finally included in the study (n=136). There were 22,611 healthy people in the control group, including incomplete data (n=1888), presence of underlying diseases such as hyperglycemia (n=11808), female (n=8284), and finally included in the study (n=631).


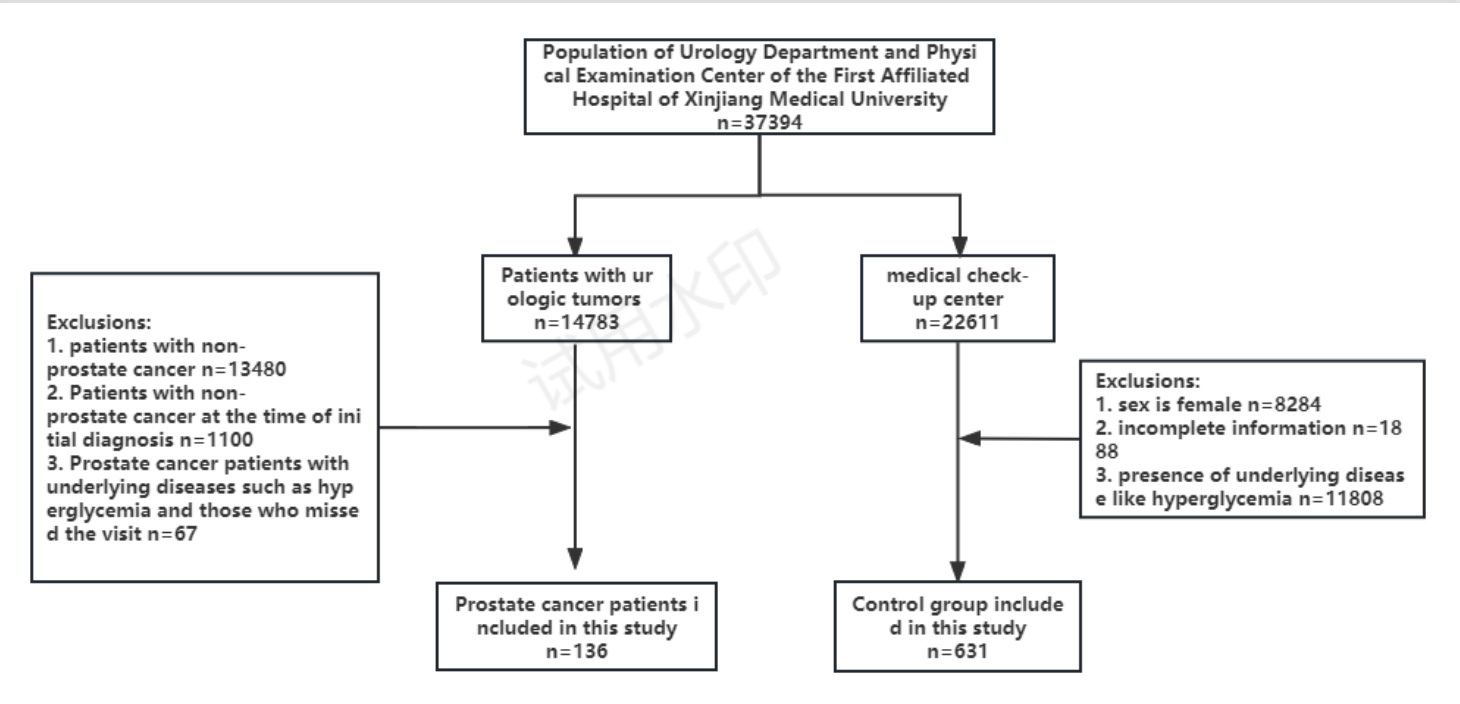

Supplement: Supplemental Information 1 [file peerj-11-16313-s001.docx]
