# Supplementary material for: Association of triglyceride-glucose index with the risk of prostate cancer: a retrospective study
Source: PeerJ. 2023 Nov 7;11:e16313. doi: 10.7717/peerj.16313 (PMC10637243; doi:10.7717/peerj.16313)

We calculated the sample size required for this case-control study by using the PASS11.0 software.Sample size N=644, N1=44 for the case group and N2=600 for the control group. By calculation, the sample size we used meets the sample requirements of this case-control study.


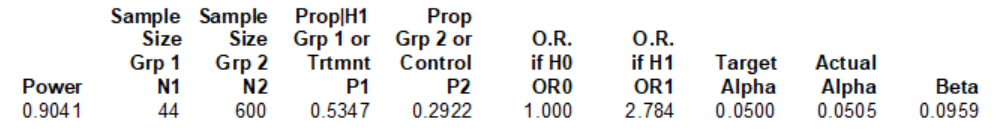

Supplement: Supplemental Information 2 [file peerj-11-16313-s002.docx]
